# Effect of chiropractic manipulative therapy on reaction time in special operations forces military personnel: a randomized controlled trial

**DOI:** 10.1186/s13063-018-3133-2

**Published:** 2019-01-03

**Authors:** James W. DeVocht, Robert Vining, Dean L. Smith, Cynthia Long, Thomas Jones, Christine Goertz

**Affiliations:** 1 Palmer Center for Chiropractic Research, Palmer College of Chiropractic, 741 Brady St., Davenport, IA 52803 USA; 2 Department of Kinesiology and Health, 26E Phillips Hall, Miami University, Oxford, OH 45056 USA; 3 Chiropractic Clinic, Blanchfield Army Community Hospital, 650 Joel Drive, Fort Campbell, KY 42223-5349 USA; 4 Present Address: The Spine Institute for Quality (Spine IQ), Davenport, IA USA

**Keywords:** Spinal manipulative therapy, Reaction time, Response time, Special Operations Forces, Military, Chiropractic

## Abstract

**Background:**

Chiropractic manipulative therapy (CMT) has been shown to improve reaction time in some clinical studies. Slight changes in reaction time can be critical for military personnel, such as special operation forces (SOF). This trial was conducted to test whether CMT could lead to improved reaction and response time in combat-ready SOF-qualified personnel reporting little or no pain.

**Methods:**

This prospective, randomized controlled trial was conducted at Blanchfield Army Community Hospital, Fort Campbell, KY, USA. Active-duty US military participants over the age of 19 years carrying an SOF designation were eligible. Participants were randomly allocated to CMT or wait-list control. One group received four CMT treatments while the other received no treatment within the 2-week trial period. Assessment included simple hand/foot reaction time, choice reaction time, and Fitts’ Law and whole-body response time. On visits 1 and 5, the same five assessments were conducted immediately pre- and post-treatment for the CMT group and before and after a 10-min wait period for the wait-list group. Primary outcomes included between-group differences for the pre-CMT/wait-list period at visit 1 and visit 5 for each test. Secondary outcomes included between-group differences in immediate pre- and post-(within visit) changes. Analysis of covariance was used for all data analysis.

**Results:**

One hundred and seventy-five SOF-qualified personnel were screened for eligibility; 120 participants were enrolled, with 60 randomly allocated to each group. Due to technical problems resulting in inconsistent data collection, data from 77 participants were analyzed for simple hand/foot reaction time. The mean ± standard deviation (SD) age was 33.0 ± 5.6 years and all participants were male. No between-group statistically significant differences were found for any of the five biomechanical tests, except immediate pre- and post-changes in favor of the CMT group in whole-body response time at both assessment visits. There were four adverse events, none related to trial participation.

**Conclusions:**

A single session of CMT was shown to have an immediate effect of reducing the time required for asymptomatic SOF qualified personnel to complete a complex whole-body motor response task. However, sustained reduction in reaction or response time from five tests compared with a wait-list control group was not observed following three sessions of CMT.

**Trial registration:**

ClinicalTrials.gov, NCT02168153. Registered on 12 June 2014.

## Background

United States military special operation forces (SOF) personnel are required to maintain high levels of physical fitness and the ability to perform activities requiring quick reactions to diverse situations, including those that can be life-threatening. Therefore, a high level of neurological function is one necessary component of maintaining the combat readiness of SOF personnel.

Efficient conscious and unconscious processing of sensory information resulting in coordinated motor responses are necessary for activities requiring prompt reactions to different sensory stimuli. Compromised sensory stimulus transmission, interpretation of such stimulus, or improper synchronization of responses can result in delayed, inaccurate, or uncoordinated reactions. Theoretically, any dysfunction within these complex neurological pathways, which may not be observable via symptoms, could lead to aberrant reactions or delayed reaction and/or response times [1].

Spinal manipulation, the primary treatment delivered by doctors of chiropractic is thought to impart some of its therapeutic benefit through several complex neurological mechanisms involving both spinal and cortical regions of the central nervous system. Research indicates that spinal manipulation causes plastic changes in sensorimotor integration within the central nervous system in human participants, particularly within the prefrontal cortex [2, 3]. Spinal manipulation appears to alter the net excitability of low-threshold motor units, increase cortical drive, and prevent fatigue [4]. These neurological mechanisms may explain improved reaction time [5], movement time [6, 7], motor control [8], and muscular strength [9] following spinal manipulation. Kelly et al. found a significant improvement in a complex reaction time task after receiving spinal manipulation [5]. Both Smith et al. [7] and Passmore et al. [6] reported that hand and head movements in response to visual stimuli were completed more quickly after participants received spinal manipulation. Daligadu et al. [10] reported results from 10 volunteers with subclinical neck pain who completed specified keypad input sequences more quickly after receiving spinal manipulation. No adverse events (AEs) were reported in any of these studies.

Although prior studies suggest that spinal manipulation can positively influence reaction and response time in the short-term, it is unknown if performance can be optimized in persons with little or no pain and with high-level motor control and coordination skills. If changes do occur, it is not yet known if multiple applications convey a cumulative effect, or if longer-term changes occur. Optimizing reaction and response time in SOF military personnel has the potential to improve performance in activities requiring rapid and accurate hand-eye coordination and instantaneous decision making leading to physical movement of the hands, arms, or legs. Improved and coordinated reactions and responses to specific stimuli can be vital during combat military operations and other activities by reducing cognitive processing time. Understanding whether spinal manipulation has such an effect in persons with high-level physical capability also sheds more light on potential mechanisms influenced by spinal manipulation. This randomized controlled trial (RCT) was conducted to answer the question: does chiropractic manipulative therapy (CMT) lead to improved reaction and response time in combat-ready SOF personnel reporting little or no pain?

## Methods

### Setting and participants

A protocol describing detailed trial methods has been previously published [11]. This prospective RCT was conducted at Blanchfield Army Community Hospital, Fort Campbell, KY, USA. Active-duty US military participants over the age of 19 years carrying the designation of SOF were eligible. The trial was approved by the following institutional review boards: Rand Corporation, Palmer College of Chiropractic, and Dwight D. Eisenhower Army Medical Center. Oversight also occurred by an independent data and safety monitoring committee. All participants provided written informed consent. Participants were not compensated for participation.

Exclusion criteria included: average pain intensity over the past week anywhere in the body rated ≥ 4 on a 0 (no pain) to 10 (worst possible pain) numerical rating scale; bone or joint pathology that constituted contraindication to receiving CMT; requiring additional diagnostic procedures; being currently treated for traumatic brain injury; pending deployment or other situations that would prevent clinic visits during the 2–4 weeks of the trial participation period; or having received CMT within the previous 30 days. Initially, the upper age limit was restricted to 45 years and SOF personnel did not include women due to technical subclassifications of SOF personnel. After 41 participants were enrolled, eligibility criteria were changed to remove the upper age limit and to allow women and those who otherwise qualified for SOF.

### Trial design

Potential participants attended an initial visit with a project manager, which comprised a consent process when all aspects of trial participation were explained. If eligible and interested in joining, participants signed an institutional review board-approved informed consent document. Following the informed consent process, participants reported demographic information and a numerical rating scale of pain intensity. The project manager then screened for nonclinical eligibility criteria. Eligible participants received an examination by one of the trial doctors of chiropractic to screen for contraindications to chiropractic care. Participants were eligible for enrollment when no contraindications were identified. At the end of the first visit, eligible participants were randomly allocated to one of two groups: CMT or wait-list control. Enrolled participants also practiced each of the five biomechanical tests (see Biomechanical tests section below).

Group assignment was performed using concealed allocation in a 1:1 ratio by a predetermined, computer-generated, restricted randomization scheme with random block sizes. The project manager, who had no knowledge of details of the randomization process, accessed a secure, web-based computer allocation module designed for this trial. Once allocated, the project manager notified participants of their group assignment.

During visit 2, all participants performed the first assessment consisting of five biomechanical tests. At the beginning of visit 2, all participants performed each of the five tests twice. Participants in the CMT group performed all five tests prior to the first treatment. After treatment, all five tests were again completed. Those in the wait-list control group did not receive CMT. Instead, wait-list group participants performed all five tests followed by a 10-min wait period before again performing each test. Participants in the CMT group received three more treatments for a total of four treatments over a 2-week period. Those in the wait-list control group did not have visits between biomechanical assessments. The testing protocol was repeated during the final visit (visit 5) and concurrent with the fourth CMT session for those in the CMT group.

The final assessment visit (visit 5) was held approximately 10 days after visit 2. Therefore, the two assessment visits for participants in both groups were separated by the same time frame. Following the final visit, those in the wait-list control group were offered the opportunity to receive four sessions of CMT over 2 weeks, just as those in the CMT group received during trial participation.

### Interventions

CMT for this trial was provided by two doctors of chiropractic serving as providers within the military treatment facility, each with more than 9 years of experience. The specific treatment given at each visit was determined individually by information obtained from clinical evaluation, which included standard orthopedic tests, range-of-motion assessments, and spinal palpatory examination. The CMT provided was composed of high-velocity, low-amplitude (HVLA) spinal manipulation procedures [12] consisting of manually applied thrusts to cervical, thoracic, or lumbo-pelvic areas when indicated by analyzing factors such as comorbid or complicating diagnoses, prior response to care (if known), local tenderness, hypertonicity, hypomobility, positions of relief and provocation, and other individual factors [11].

### Biomechanical tests

Five tests measured reaction or response time. Reaction time was defined as the length of time occurring between a prompt and the first body movement in response to the prompt. Reaction time tests were modeled after those reported by Luoto et al. [13]. Response time was defined as the duration of time occurring between a prompt and accomplishment of a task involving both reaction and movement time [7].

#### Simple reaction time of the dominant hand

Participants sat in front of a computer screen holding an electronic switch in their dominant hand. When a prompt appeared on the screen, participants pressed a switch with the thumb of their dominant hand. The test consisted of a series of 11 prompts, with the results from the first prompt being discarded. The time between a button press for one prompt and the appearance of the next prompt randomly varied from 0.5 to 4 s.

#### Simple reaction time of the dominant foot

This test was conducted in the same manner as the reaction time test for the dominant hand except participants pressed an electronic pedal switch with the dominant foot upon recognizing the computer screen prompt.

#### Choice reaction time

Participants again sat before a computer screen holding an electronic thumb switch in each hand. Each foot rested on an electronic pedal switch. Prompts appeared on the screen specifying which button or pedal to press. For this test, a constant 1-s delay separated the press of a button or pedal and the appearance of the next prompt. This test consisted of a series of 41 prompts. Response to the first prompt was not included in the results.

#### Response time

**Fig. 1:**
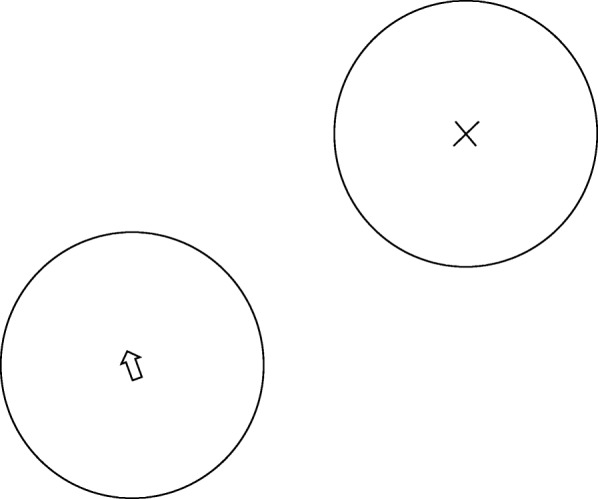
Depiction of computer image used for the Fitts’ law test. The curser is moved using a computer mouse from one circle to the other and clicked by a participant. The curser is then moved back to the original circle and clicked again, finishing the sequence. Thirty-two sequences using pairs of differently oriented and sized circles were used

Fitts’ Law was used with methods drawn from Smith et al. [7]. Participants again sat in front of a computer screen. This test consisted of a series of pairs of circles. Each time a pair of circles appeared on the screen, one circle included the letter X while the other contained the computer cursor (Fig. 1). Participants, using the mouse, moved the cursor from one circle to the circle with the X. Once inside, the mouse was clicked. Immediately upon the click, the X disappeared from that circle, appearing instead in the circle where the cursor was originally located. Participants then moved the cursor back to the original circle and clicked the mouse, completing the sequence. When ready to continue, participants clicked anywhere on the blank screen, which caused the next pair of circles to appear and reactivated the electronic timer. Timing for the test was recorded only when circles were visible on the computer screen. Participants took as much time as needed between each sequence. The test consisted of a series of 32 circle pair sequences. Circles within each pair were identical in size, but different pairs were oriented differently with variably sized circles. The distance between all circle centers for each test pair was identical.

#### Response time (whole body)

**Fig. 2:**
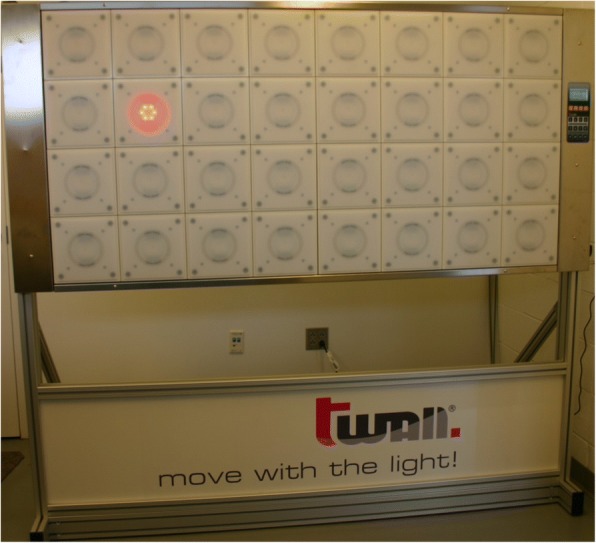
Image of the t-wall®. Participants manually strike the lit panel, causing another panel to light in random sequence. Participants completed this test as quickly as possible by striking a random sequence of 100 lit panels

The t-wall® (Motion Fitness, Rolling Meadows, IL, USA) is a commercially available device consisting of a panel of 32 touch pad lights (Fig. 2). Each panel is 8 × 8 inches arranged in a 4 × 8 foot array. Participants stand in front of the wall with one touch pad lit. The test begins when the pad that is hit lightly with the hand, which turns off the light and immediately lights another panel. The test consists of hitting a series of 100 consecutively lit touch pads in random sequence. Timing for this test begins when the participant hits the first lit touch pad and ends when the last panel in the sequence is hit. Participants were encouraged to complete each test as quickly as possible. Two random sequences were used for each test to prevent anticipation and a learning effect. Each of the five tests used in this trial are described in greater detail in the trial protocol [11]. Data management personnel responsible for processing raw data were blinded to group assignment.

### Statistical analysis

An intention-to-treat approach was used in which participants were analyzed according to their original treatment allocation. Data analyses were performed using SAS (version 9.4, Cary, NC). Because age eligibility changed mid-way through the trial, age was controlled for in the data analyses. The primary analyses compared the mean changes in reaction and response time from sequence A, performed before the CMT/break at visit 2, to sequence A performed before the CMT/break at the final visit (visit 5) between the treatment and wait-list control groups, using an analysis of covariance controlling for age for each of the five biomechanical tests. We did not control for analyzing multiple outcomes. Between-group mean differences from the analysis of covariance were reported, adjusted for age, with 95% confidence intervals. The level of significance was set at 0.05. The secondary analyses compared the immediate changes in sequence A before the CMT/break to sequence A after the CMT/break at both visit 2 and the final visit using the same methods described above.

### Sample size

The sample size for this trial was based on a power analysis that used the standard deviations (SDs) of mean changes in reaction and response times over a 1-week period for each of the five biomechanical variables obtained in a pilot study. The calculations were based on an estimated effect size of a 10% change after CMT of the mean reaction or response time as measured in the first assessment for each variable with the assumption that the wait-list control group would have no change. Fifty participants per group would have at least 85% power to detect a 10% or larger difference at a 0.05 level of significance in mean change between groups. To account for the possibility of a 15% loss to follow-up, we increased the total sample size to 120, with 60 per group.

## Results

**Fig. 3:**
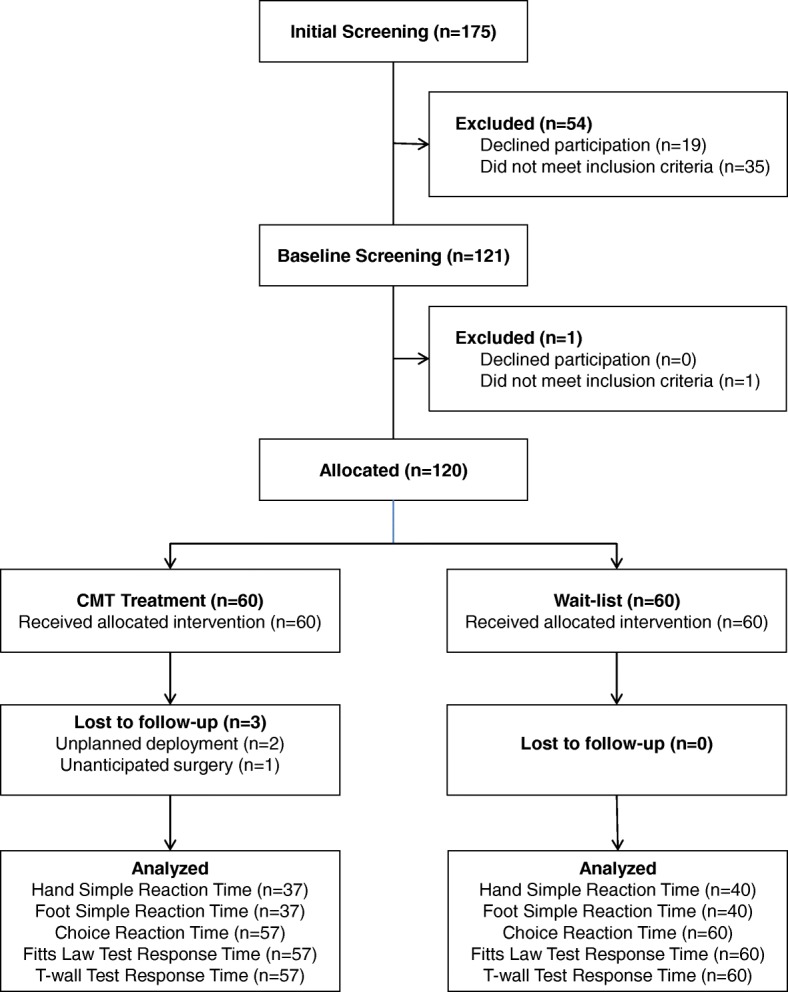
Trial flow diagram. CMT chiropractic manipulative therapy

During the enrollment period, 175 SOF-qualified individuals were screened for eligibility as shown in the trial flow diagram (Fig. 3). Of those, 55 were excluded, resulting in 120 participants. The first participant was enrolled on 30 September 2014 and data collection was completed on 7 June 2016. The two most common reasons for exclusion were not being SOF qualified and scheduling conflicts. Participants were primarily referred to the project manager from physical therapists (49) and other healthcare providers (38). The remainder of the participants were recruited from informational presentations by the site project manager (24), informational emails (6), and the SOF newsletter (3). Adverse events were defined as any unexpected or unusual medical occurrence during the conduct of the trial that may or may not have a causal relationship with trial procedures [14].

Four adverse events were reported during the course of this trial. None were related to trial procedures. Two participants suffered minor knee injuries, one while running down stairs (meniscal inflammation) and another during a military training exercise (patellar bruise). One participant reported upper back pain while exercising at a gym. Another reported aggravation of a pre-existing umbilical hernia while exercising at a gym. No adverse event was severe enough to restrict participation in the trial. All adverse events were evaluated and managed by nontrial-related military healthcare providers.

### Baseline characteristics

Table 1 displays baseline characteristics. The mean ± SD age of participants was 33.0 ± 5.6 years and all participants were male. Characteristics were similar between groups.

**Table 1:** Baseline characteristics

	CMT (*n* = 60)		Wait-list controls (*n* = 60)	
Age (years), mean (SD)	32.8	(5.1)	33.2	(6.1)
Race, *n* (%)				
Black or African American	3	(5)	2	(3)
White	54	(90)	55	(92)
Other	3	(5)	3	(5)
BMI (kg/m^2^), mean (SD)	27.8	(2.7)	27.3	(2.7)
Pain intensity, median (range)	2.0	(0–3.0)	2.0	(0–3.0)

*BMI* body mass index, *CMT* chiropractic manipulative therapy, *SD* standard deviation

### Trial outcomes

Table 2 contains the results of the five biomechanical outcome measures obtained from the two different assessment sessions. Reaction time tests are displayed as mean duration for participants reacting to a single prompt. The response times shown (Fitts’ Law and t-wall®) represent the total time needed to complete the entire sequence of prompts for each test. There are additional missing data for the simple hand and simple foot reaction tests due to a problem in early data collection. The connection between the computer software program (Paradigm software package, Perception Research Systems, Inc.) and the MP150 Data Acquisition System (BIOPAC Systems, Inc.) was reset inconsistently after each response resulting in inaccurate reaction time computation. Once identified, the problem was corrected. Because data collected before the technical issue was corrected were unreliable and likely inaccurate, they were not included in the analysis for these two measures. There were no statistically significant between-group differences during this time period for any of the five tests.

**Table 2:** Outcome variables taken just before treatment or break during visit 2 and during final visit, with changes between visits and group differences

	CMT				Wait-list control				Change from visit 2 to final visit				Between group differences (95% CI)	*P* value
	Visit 2		Final visit		Visit 2		Final visit		CMT		Control			
Hand simple reaction time (ms)*	258.7	(48.6)	252.6	(33.7)	254.7	(46.4)	252.3	(49.3)	−4.88	(42.57)	−1.68	(53.56)	−3.49 (−25.75 to 18.77)	0.76
Foot simple reaction time (ms)*	291.9	(41.8)	300.6	(32.1)	300.6	(41.8)	307.0	(49.3)	8.65	(39.98)	7.88	(42.83)	0.97 (− 18.04 to 19.98)	0.92
Choice reaction time (ms)**	426.1	(56.2)	421.7	(55.7)	445.9	(72.9)	436.4	(67.9)	−6.42	(44.06)	−9.51	(53.29)	3.49 (−14.40 to 21.39)	0.70
Fitts’ Law test response time (s)**	65.9	(0.40)	66.3	(0.72)	67.1	(0.66)	66.6	(0.32)	0.412	(3.710)	−0.548	(3.731)	0.988 (−0.373 to 2.349)	0.15
t-wall® response time (s)**	49.1	(4.7)	47.2	(4.2)	50.1	(4.2)	48.5	(4.4)	−2.00	(2.40)	−1.59	(2.10)	−0.41 (−1.24 to 0.41)	0.32

Values are shown as mean (standard deviation) based on analysis of covariance (ANCOVA) model, adjusted for age

*CI* confidence interval, *CMT* chiropractic manipulative therapy

*n = 37 in CMT and *n* = 40 in wait-list control

**n = 57 in CMT and *n* = 60 in wait-list control

Tables 3 and 4 display immediate changes in the five biomechanical tests performed prior to receiving CMT or the 10-min wait period during the first assessment and second assessment, respectively. The CMT group experienced a larger reduction in t-wall® response time following treatment than the wait-list group at both assessments (*P* = 0.03). No statistically significant between-group differences were observed for any of the other tests in either assessment session.

**Table 3:** Immediate changes in pre- and post-reaction and response time (visit 2)

	CMT	Wait-list control	Mean between group difference (95% CI)	*P* value
Hand simple reaction time (ms)**	−1.13 (48.16)	14.25 (37.75)	−15.92 (−35.35 to 3.51)	0.11
Foot simple reaction time (ms)**	1.52 (38.10)	−2.47 (37.23)	4.20 (−12.81 to 21.21)	0.62
Choice reaction time (ms)***	−13.03 45.12)	−13.49 (36.32)	0.23 (−14.59 to 15.06)	0.98
Fitts Law test response time (s) ***	−0.86 (2.46)	−1.40 (2.77)	0.54 (−0.41 to 1.49)	0.27
t-wall® test response time (s)***	−0.30 (2.25)	0.59 (2.20)	−0.90 (−1.71 to −0.09)	0.03

Values are shown as mean (standard deviation) based on analysis of covariance (ANCOVA) model, adjusted for age

*CI* confidence interval, *CMT* chiropractic manipulative therapy

**n = 39 in CMT and *n* = 40 in wait-list control

***n = 60 in CMT and *n* = 60 in wait-list control

**Table 4:** Immediate changes in pre- and post-reaction and response time (final visit)

	CMT	Wait-list control	Mean between group difference (95% CI)	*P* value
Hand reaction time (ms)**	4.13 (39.17)	15.04 (52.08)	−10.63 (−31.34 to 10.08)	0.31
Foot reaction time (ms)**	5.57 (47.52)	6.93 (38.12)	−1.53 (−20.79 to 17.74)	0.88
Choice reaction time (ms)***	−5.33 (35.90)	−13.47 (40.23)	8.31 (−5.71 to 22.34)	0.24
Fitts’ Law test response time (s) ***	−2.06 (2.20)	−1.38 (2.42)	−0.64 (−1.47 to 0.18)	0.13
t-wall® test response time (s)***	−0.46 (1.77)	0.28 (1.95)	−0.75 (−1.43 to −0.06)	0.03

Values are shown as mean (standard deviation) based on analysis of covariance (ANCOVA) model, adjusted for age

*CI* confidence interval, *CMT* chiropractic manipulative therapy

**n = 39 in CMT and *n* = 41 in wait-list control

***n = 57 in CMT and *n* = 60 in wait-list control

## Discussion

To our knowledge this trial is the first to explore reaction and response time in US military SOF personnel following a short course of CMT. We found no statistically significant differences in reaction or response time over the trial period, which lasted approximately 2 weeks. Response time measured with the t-wall® showed a significant difference in immediate (pre- and post-) changes between groups, observed during both assessments. There were no statistically significant between-group differences in immediate pre- and post-measures for the other four biomechanical tests.

In general, findings are inconsistent with the research literature that reports improved reaction and response time following spinal manipulation [5–8]. This apparent inconsistency may be due to the high-level physical conditioning characteristic of SOF personnel. In such individuals, neuromuscular systems already function at optimum or near-optimum levels and further improvement in reaction or response time may not be possible, regardless of intervention. This trial did not assess whether reaction or response time for non-SOF-qualified individuals or those with musculoskeletal symptoms are influenced by CMT. The trial is limited by the loss of data due to inaccurately computed simple hand and foot reaction times for a subset of participants. Although results could possibly be different if data from the entire sample were consistently collected and analyzed, findings are consistent with results from the other three assessments in this trial. Therefore, it seems unlikely that the lower number of participants analyzed for these two tests had much influence on overall findings.

Of the five tests used in this trial, the t-wall® test measured the longest continuous response time, lasting approximately 1 min. The immediate performance improvement in the CMT group at both visits observed in this trial suggests that CMT may influence complex tasks that require longer continuous periods of time to complete, suggesting the need for further research in this area to understand the underlying neuromuscular mechanism(s) at work. Similarly, it is possible that conducting Fitts’ Law tests with more trials, or possibly trials with smaller circles making it more difficult, might enable discernment of differences between groups.

## Conclusions

A single session of CMT was shown to have an immediate effect of reducing the time required for asymptomatic SOF-qualified personnel to complete a complex motor response task. However, three sessions of CMT did not induce sustained reduction in reaction or response time associated with five different biomechanical tests compared with a wait-list control group.

## Abbreviations

AE: Adverse event
CMT: Chiropractic manipulative therapy
HVLA: High-velocity, low-amplitude
RCT: Randomized controlled trial
SD: Standard deviation
SOF: Special operations forces
